# Hints for Genetic and Clinical Differentiation of Adult-Onset Monogenic Autoinflammatory Diseases

**DOI:** 10.1155/2019/3293145

**Published:** 2019-12-31

**Authors:** Carla Gaggiano, Donato Rigante, Antonio Vitale, Orso Maria Lucherini, Alessandra Fabbiani, Giovanna Capozio, Chiara Marzo, Viviana Gelardi, Salvatore Grosso, Bruno Frediani, Alessandra Renieri, Luca Cantarini

**Affiliations:** ^1^Clinical Pediatrics, Department of Molecular Medicine and Development, University of Siena, Siena, Italy; ^2^Institute of Pediatrics, Fondazione Policlinico A. Gemelli IRCCS, Rome, Italy; ^3^Periodic Fever Research Center, Università Cattolica Sacro Cuore, Rome, Italy; ^4^Research Center of Systemic Autoinflammatory Diseases and Behçet's Disease Clinic, Department of Medical Sciences, Surgery and Neurosciences, University of Siena, Siena, Italy; ^5^Medical Genetics, University Hospital of Siena, Siena, Italy

## Abstract

Monogenic autoinflammatory diseases (mAIDs) are inherited errors of innate immunity characterized by systemic inflammation recurring with variable frequency and involving the skin, serosal membranes, synovial membranes, joints, the gastrointestinal tube, and/or the central nervous system, with reactive amyloidosis as a potential severe long-term consequence. Although individually uncommon, all mAIDs set up an emerging chapter of internal medicine: recent findings have modified our knowledge regarding mAID pathophysiology and clarified that protean inflammatory symptoms can be variably associated with periodic fevers, depicting multiple specific conditions which usually start in childhood, such as familial Mediterranean fever, tumor necrosis factor receptor-associated periodic syndrome, cryopyrin-associated periodic syndrome, and mevalonate kinase deficiency. There are no evidence-based studies to establish which potential genotype analysis is the most appropriate in adult patients with clinical phenotypes suggestive of mAIDs. This review discusses genetic and clinical hints for an ideal diagnostic approach to mAIDs in adult patients, as their early identification is essential to prompt effective treatment and improve quality of life, and also highlights the most recent developments in the diagnostic work-up for the most frequent hereditary periodic febrile syndromes worldwide.

## 1. Introduction

Monogenic autoinflammatory diseases (mAIDs) are clinical entities characterized by recurrent inflammatory attacks occurring without any evidence of infections, neoplasms, or deregulation of the adaptive immune system. This expanding family of diseases is actually known to be caused by mutations in genes involved in the regulation of innate immunity, inflammation, and cell death, including first-line responses to infectious agents and different tissue injuries [1]. Mutations in the *MEFV* gene were firstly identified for patients with familial Mediterranean fever (FMF) in 1997 [2, 3]. Few years later, the genetic basis of three other mAIDs was detected through candidate gene approach, linkage analysis, and/or homozygosity mapping. Familial Hibernian fever, commonly known as tumor necrosis factor receptor-associated periodic syndrome (TRAPS), was found to be caused by mutations in the *TNFRSF1A* gene [4]. In 1999, Drenth et al. identified mutations in the gene encoding mevalonate kinase (*MVK*), the key enzyme in isoprenoid and sterol synthesis, which is involved in the pathogenesis of mevalonate kinase deficiency (MKD) and hyperimmunoglobulinemia D syndrome (HIDS) [5]. Furthermore, gain-of-function mutations in the *NLRP3* gene (also known as *CIAS1*) were associated with the disease spectrum of cryopyrin-associated periodic syndrome (CAPS) in 2001 [6, 7].

Over the last 20 years, the identification of new putative genes following the extensive use of next-generation sequencing technologies and the remarkable progress of molecular techniques have deepened our knowledge about the pathogenesis of mAIDs, providing novel insights of mechanisms involved in innate immunity regulation [8]. In addition, the discovery of gene modifiers and somatic mosaicisms as well as a better comprehension of the role of epigenetic factors have clarified some aspects of the wide phenotypic heterogeneity of these disorders.

Although recurrent high-grade fever represents a common ground for most mAIDs, the clinical presentation may be variable, being all organs and tissues potentially targeted by inflammation. Moreover, atypical or oligosymptomatic presentations are not rare, especially in patients with adult-onset disease [9, 10]. In this regard, recent studies have proved that mAIDs may start off not only in the pediatric age but also during adulthood [11–14]. A delayed onset of mAIDs is often due to low-penetrance mutations which may be sometimes identified even in healthy carriers [15–18]. For these reasons, early identification of probands, correct interpretation of low-penetrance mutations, and prevention of overdiagnosis and overtreatment can be challenging [19, 20]. New evidence-based classification criteria for hereditary recurrent febrile syndromes have been recently developed on the basis of international expert consensus and evaluated in a large cohort of patients from the Eurofever registry in 2019 with the aim of recruiting patients for translational and clinical studies, and not misused as diagnostic criteria [21].

This review is aimed at describing genetic and clinical clues of the four historical mAIDs in order to suggest an empirical flow chart for diagnosis. Single organ involvement and systemic features will be examined, with particular attention to differential diagnosis with multifactorial AIDs. In addition, the description of the most frequently identified mutations and an overview of genetic data will be provided to facilitate physicians unravel among pathogenic variants, polymorphisms and mosaicisms for genes related to mAIDs. Genetic and clinical features of the four historical mAIDs are summarized in the Table 1.

An extensive literature search in the Medline database (via Pubmed) was performed up to May 2019. We searched for studies through the following words: “monogenic autoinflammatory disease”, “familial Mediterranean fever”, “mevalonate kinase deficiency”, “tumor necrosis factor receptor-associated periodic syndrome”, “cryopyrin-associated periodic syndrome”, and their synonyms. Papers published in English language over the last ten years were screened for eligibility, based on title, abstract, and keywords. Papers were included if clinical clues to the diagnosis of the four mAIDs, both in children and in adults, were reported. References in the relevant papers were also reviewed. Main reports published before the aforementioned period of time were included as well.

## 2. Diagnostic and Genetic Overview of mAIDs

### 2.1. Familial Mediterranean Fever (FMF)

FMF is the most frequent and best characterized autosomal recessive monogenic AID. Southern European, Northern African, Turkish, and Arabic people are more frequently affected (the prevalence is 1 : 150-1 : 1000 in Turkey) [22]; the Middle East and Eastern Europe show a lower prevalence (1 : 10,000,000). Moreover, a growing number of FMF diagnosis were recently established in Japan, USA, and Brazil [2, 3, 23, 24]. Employing positional cloning, genomic sequence analysis, and exon trapping in 1997, two independent FMF consortia (French FMF Consortium and International FMF Consortium) identified and isolated the *MEFV* gene, located on chromosome 16p13.3, as the causative for FMF [2, 3]. This gene encodes a 781-amino acid protein known as pyrin/TRIM20/marenostrin, which works as a key component of the innate immune system and is expressed by neutrophils, eosinophils, monocytes, and dendritic cells [25]. Although characterized by an autosomal recessive pattern of inheritance, the FMF phenotype has been observed also in heterozygous patients, in whom hypothetical modifier genes and/or environmental factors may play a substantial role in inducing inflammatory attacks [26, 27]. Disease onset occurs before the age of 10 in more than 60% of patients and before the age of 30 in 98% of cases [28]. Acute febrile attacks usually last a few hours to 3 days; serositis, articular symptoms, and erysipelas-like erythema in the lower limbs are the most typical manifestations accompanying fever. Although adult-onset patients often manifest a milder phenotype, clinical features are generally similar to those expressed by younger patients, except for a lower frequency of arthritis and skin erythema [29]. Systemic reactive AA amyloidosis represents the most severe long-term complication in untreated FMF patients [30]. In this regard, three different FMF types have been suggested: type 1 FMF refers to the presence of overt clinical inflammatory disease; type 2 FMF presents with systemic amyloidosis in otherwise asymptomatic subjects; and type 3 FMF is related to the absence of inflammatory manifestations and systemic amyloidosis in subjects carrying *MEFV* mutations [31].

Since the identification of the *MEFV* gene, more than 340 nucleotide variants have been found, half of them being clearly associated with FMF. The majority of FMF-causing mutations are located within exon 10 and are mutational hotspots (including p.M694V/I and p.M680I), which are associated with a more severe clinical phenotype. Milder pathogenic variants, such as p.V726A located on exon 10, have been also reported [23, 32–34]. Moreover, other mutations, either of unknown or uncertain significance (p.K695R, p.P369S, p.F479L, p.I591T, and p.E148Q) and pathogenic variants (p.R761H, p.A744S, p.I692del, p.E167D, and p.T267I), have been associated with varying degrees of disease severity [35, 36]. Despite extensive studies over the last 20 years, genotype-phenotype correlations in FMF have not been fully understood [37]. Many patients with clinical FMF have no genetic variants or are heterozygous for *MEFV*, highlighting the possibility of an autosomal-dominant transmission or of a clinical expression depending on additional modifying factors as modifier genes and environmental or epigenetic influences. Regarding FMF patients with a single heterozygous mutation (such as p.H478Y, p.T577S/A/N, p.M694del/I, p.E148Q, and p.L110P), they were associated with a wide clinical variability, including an incomplete or less severe disease phenotype [38–40]. Moreover, modifier genes encoding components of amyloid A deposits, such as serum amyloid A1 (*SAA1*), have been significantly and independently associated with renal amyloidosis [41]. In addition, peripheral leukocytes from FMF patients have shown reduced *MEFV* transcript expression due to a slightly increased methylation of exon 2 compared to healthy controls [42]. Among epigenetic modifications, a differential expression of several miRNAs has been demonstrated both in homozygote and heterozygote quiescent FMF patients, compared to controls and healthy carriers [43–46].

Pyrin is a member of the TRIM protein family playing a pivotal role in the inflammatory response against infections through the regulation of interleukin- (IL-) 1*β* production [47]. The protein is formed by N-terminal pyrin domain (PYD), zinc finger domain (bBox), coiled-coil (CC), and B30.2/SPRY C-terminal domain: the pathogenetic mechanism which links *MEFV* gene mutations to the development of the FMF phenotype is not fully clarified. According to Papin et al., pyrin SPRY domain interacts with inflammasome components inhibiting pro-IL-1*β* processing; this C-terminal region of the protein is frequently altered as a consequence of pathogenic *MEFV* mutations [48]. Consequently, macrophages from pyrin knock-out mice show enhanced IL-1*β* release in response to inflammatory stimuli [49]. On the contrary, Chae et al. demonstrated that pyrin mutations in FMF-knock-in (KI) mice induce NLRP3-independent IL-1*β* activation. In fact, macrophages obtained from KI FMF-associated B30.2 mutations mice (including M680I, M694V, and V726A) showed IL-1*β* overproduction after lipopolysaccharide (LPS) stimulation, while no reduction of IL-1*β* production was observed in NLRP3-deficient KI mice (*MEFV*^V726A/V726A^*NLRP3*^−/−^). These findings suggested that gain-of-function pyrin mutations induce IL-1*β* activation through an NLRP3-independent manner. Conversely, monocytes from patients with FMF had LPS-induced IL-1*β* oversecretion, which was damped after in vitro NLRP3 downregulation [50]. Interestingly, the same authors observed that hyperproduction of IL-1*β* correlated with both number and penetrance of *MEFV* mutations. In particular, individuals carrying heterozygous p.M694V or p.K695R mutations have shown higher levels of IL-1*β* release than healthy controls, but lower levels than FMF patients who were homozygous for the same mutations. In addition, silencing NLRP3 expression led to the inhibition of IL-1*β* secretion, suggesting that FMF-associated mutations might also trigger NLRP3-dependent inflammatory response [51]. More recently, the identification of biochemical processes triggering IL-1*β* production in response to bacterial modification of the GTPase RhoA shed light on the outstanding role of pyrin in the inflammatory responses. In this model, pyrin is kept inactive by serine-threonine kinases PKN1 and PKN2, through Ser208/Ser242 phosphorylation and subsequent binding to 14-3-3 proteins, which block the pyrin inflammasome. It has been demonstrated that PKN1 and PKN2 are activated by RhoA GTPase and that Rho-modifying bacterial toxins induce inactivation of these kinases, resulting in pyrin-inflammasome formation and IL-1*β* production. Interestingly, mutated pyrin from FMF patients displayed a low binding to 14-3-3 proteins and PKN [52]. The latest research on pyrin functions focused on its interaction with the cytoskeletal network, demonstrating its pivotal role in modulating inflammatory cell polarization and migration. Supporting these new data, bioinformatics analyses revealed that mRNAs targeted by miRNAs which are found overexpressed in homozygous FMF patients (such as miR-20a-5p and miR-197-3p) cluster in inflammatory pathways related to cell migration [53].

**Figure 1:**
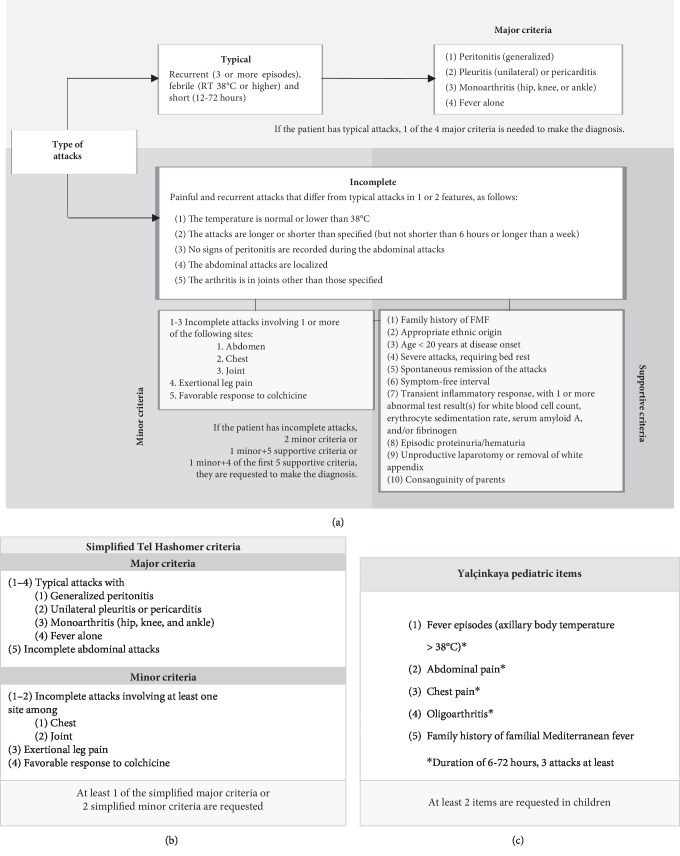
Complete (1a) and simplified (1b) Tel Hashomer criteria adapted by from Livneh et al. [54] for the diagnosis of familial Mediterranean fever (FMF); Yalçinkaya items (1c) for the diagnosis of FMF in childhood, adapted from Yalçinkaya et al. [55].

FMF diagnosis does not necessarily require genetic analysis, as it is also driven by clinical evaluation and relies on diagnostic criteria [54–56]. Several sets of criteria have been suggested over time. However, those established at the Tel Hashomer Medical Center in Israel are currently the most widely used for adult patients [54]. They include two sets of diagnostic criteria, consisting of a complete version and a simplified one: the complete version classifies FMF clinical manifestations into major and minor items and also includes ten supportive criteria, as illustrated in Figure 1(a). Both specificity and sensitivity of these criteria are higher than 95% [54]. The Tel Hashomer group also proposed simplified criteria that include major and minor criteria, as shown in Figure 1(b). In this case, FMF diagnosis is allowed when at least 1 major criterion or at least 2 minor criteria are fulfilled. The simplified version includes atypical attacks with abdominal involvement as a major criterion. Sensitivity and specificity are higher than 95% for simplified criteria as well [54]. More recently, Yalçinkaya et al. have proposed diagnostic criteria specifically tailored for children, as described in Figure 1(c) [55].

### 2.2. Tumor Necrosis Factor Receptor-Associated Periodic Syndrome (TRAPS)

Originally described in 1982 in a family of Irish/Scottish descent and previously named “Hibernian fever” from the ancient Latin name of Ireland *Hibernia*, TRAPS is caused by mutations in the *TNFRSF1A* gene, inherited in an autosomal-dominant pattern [57–59]. At the end of the ‘90s, genome-wide association studies and linkage analysis in affected families placed the susceptibility locus on the distal short arm of chromosome 12p13, encoding tumor necrosis factor (TNF) receptor type 1 (TNFRSF1A/TNFR1/p55/CD120a) [58]. Afterwards, McDermott et al. identified germline mutations in the *TNFRSF1A* gene in seven families of Irish, Scottish, English, German, Finnish, and French-Canadian ancestry [4]. Nowadays, TRAPS is considered a rare disease, with an estimated prevalence of about 1 : 1,000,000 people. Most patients are of European origin, though some patients from Africa and Asia have been reported as well [60].

TRAPS is the most variable and protean among mAIDs in terms of age at disease onset, disease severity, and clinical presentation. Nevertheless, the disease is generally characterized by long-lasting inflammatory attacks that may even reach several weeks of duration. In the clinical practice, high-grade fever, erythematous migratory skin plaques associated with underlying myalgia, and joint and ocular inflammatory signs are prominent manifestations of TRAPS [60, 61]. Although most patients experience an early disease onset, adult-onset TRAPS has been widely reported, especially in subjects carrying low-penetrance mutations and presenting with atypical features, such as inflammatory manifestations affecting unusual sites [62–67]. To date, no clinical diagnostic criteria have been proposed for the diagnosis of TRAPS, which is based on the identification of *TNFRSF1A* mutations.

TNFRSF1A is a member of the TNF receptor superfamily, constitutively expressed on most cell types, which is composed of an extracellular domain consisting of the tandem repeat of four cysteine-rich domains (CRD1-4), a transmembrane region, and an intracellular death domain (DD) involved in intracellular signal transduction. The extracellular domain is characterized by the presence of intramolecular disulfide bonds, which bind TNF-*α* and also mediate the self-assembly of the receptor itself. Upon activation by a TNF ligand, TNFR1 signal transduction leads to several biochemical processes, including the release of proinflammatory cytokines through the nuclear factor-*κ*B (NF-*κ*B) pathway or, alternatively, through other intracellular cascades, such as p38 and c-Jun N-terminal kinase/mitogen-activated protein kinase (JNK/MAPK) signaling, and programmed cell death through the activation of cysteine proteases, named caspases. Moreover, activated TNFR mediates the release of the extracellular domain of the receptor itself into the extracellular compartment by means of ADAM17 (TNF-*α* converting enzyme), a key mediator of this “shedding” process, which generates a pool of sTNFR1 able to bind circulating TNF-*α*, dampening acute inflammation [62, 68].

Currently, more than 150 *TNFRSF1A* gene mutations have been identified. The majority of disease-associated mutations are missense mutations located in exons 2, 3, and 4, encoding the extracellular domain of the mature TNFR protein [69]. As previously written, low-penetrance variants are associated with milder phenotypes, later onset of disease, and lower risk of AA amyloidosis, if compared to high-penetrance mutations. Among low-penetrance variants, p.R92Q and p.P46L represent the most common mutations found in TRAPS patients; nevertheless, 2% allele frequency is estimated for p.R92Q in Caucasians, while about 10% allele frequency is estimated in the African population for p.P46L [70–74]. On the contrary, high-penetrance “structural” mutations are responsible of severe phenotypes, characterized by early onset, high frequency of disabling inflammatory attacks, and a higher risk of developing AA amyloidosis over time. Cysteine residue substitutions (i.e., p.C30R, p.C33Y, p.C43G, p.C52Y, and p.C55Y) and p.T50M are listed among the structural variants [30, 70–74]. Moreover, a novel in-frame deletion of 24 nucleotides (c.255_278del) in exon 3 was recently identified in one patient [75].

Several pathogenetic mechanisms have been studied *in vitro* to demonstrate how *TNFRSF1A* mutations contribute to the development of the TRAPS phenotype: (i) impaired TNFRSF1A cell surface expression; (ii) altered TNF-*α* binding; (iii) defective shedding of the receptor; and (iv) intracellular accumulation of mutated TNFRSF1A proteins [76–81]. The identification of reduced serum levels of soluble TNFR1 paved the way for the “defective shedding hypothesis.” Indeed, activated leukocytes carrying p.C52F, p.C33Y, p.T50M, and p.C88R mutations showed increased TNFR1 membrane expression and, on the other hand, reduced cleavage of the receptor [4]. Interestingly, further studies have demonstrated that intracellular accumulation of misfolded TNFRSF1A proteins increases oxidative stress levels, stimulating proinflammatory signaling pathways [82–85]. In support of this suggestion, chronic oxidative stress as well as enhanced IL-6 and TNF-*α* levels were observed, in response to LPS, in monocytes isolated from patients with *TNFRSF1A* structural mutations (i.e., p.C33Y, p.T50M, p.C33G, p.C52F, and p.C30Y) in comparison with healthy controls [82, 83]. Moreover, autophagy defects and endoplasmic reticulum stress with consequent X-box binding protein 1 (XBP1) hyperactivation were also proposed as pathogenetic determinants in association with TRAPS high-penetrance variants [84, 85]. Interestingly, Pucino et al. observed for the first time a T cell response pattern of activation in TRAPS patients carrying high-penetrance variants compared to healthy controls and, specifically, a lower frequency of peripheral regulatory T cells, a defective suppressive phenotype associated to ERK1/2, STAT1/3/5, mammalian target of rapamycin, and NF-*κ*B pathways [86]. Also epigenetic mechanisms have been recently added to TRAPS pathophysiology: in fact, TRAPS patients carrying structural mutations may display a specific serum signature by miRNAs. Moreover, a different expression of specific miRNAs, involving miR-92a-3p and miR-150-3p, has been observed between patients treated with the IL-1 receptor antagonist anakinra and nontreated patients [87]. More recently, Harrison et al. reported that primary dermal fibroblasts from patients carrying the p.T50M, p.C472, and p.C88R mutations showed an impaired regulation of miR-146a and miR-155, which led to increased responsiveness to LPS [88].

### 2.3. Cryopyrin-Associated Periodic Syndrome (CAPS)

CAPS is a spectrum of diseases caused by mutations in the *NLRP3* gene and including familial cold autoinflammatory syndrome (FCAS), Muckle-Wells syndrome (MWS), and chronic infantile neurological cutaneous and articular syndrome (CINCA), the latter also known as “neonatal onset multisystem inflammatory disease” or NOMID. These three clinical entities range from the least to the most severe phenotype, respectively. In particular, FCAS is characterized by recurrent attacks typically induced by generalized cold exposure and consisting of fever, urticaria-like rash, asthenia, conjunctivitis, and arthralgia; in addition to the clinical picture described for FCAS, MWS is also characterized by arthritis, optic nerve head swelling, and inflammation of the vestibule-cochlear nerve, which might lead to progressive visual loss and bilateral sensorineural hearing loss, respectively [6, 7, 89–91]. Early central nervous system involvement with chronic aseptic meningitis, increased intracranial pressure, cerebral atrophy, ventriculomegaly, and severe chronic papilledema are further identified in patients with CINCA/NOMID [92, 93]. Moreover, severe chronic arthritis with conspicuous structural deformities of large joints, bony overgrowth, and loss of articular function are typical manifestations of CINCA/NOMID [94, 95].

CAPS are rare diseases with a prevalence of 1-2 per 10^6^ in the United States and 1/360,000 in France, without any gender predilection [96]. Patients are often of Caucasian origin, and disease prevalence seems to be higher for FCAS and MWS in the US and Europe, respectively, while CINCA/NOMID is rarer [96]. The gene responsible for these syndromes was identified at the end of the 20th century after linkage analysis studies on different families of patients showing clinical CAPS pictures. Cuisset et al. applied a genome-wide search strategy to three families, mapping a region closely related to the MWS phenotype within region 44 of chromosome 1 [97]. Afterwards, mutations in the *NLRP3* gene located on chromosome 1q44 were associated to three FCAS and one MWS families and to several CINCA patients [6, 7].

Diagnosis of CAPS relies on genetic research for *NLRP3* mutations, though general clinical diagnostic criteria for CAPS have been recently developed: these criteria included the increase of inflammatory markers as a mandatory item and six other CAPS typical signs as additional items. Diagnosis should require the fulfillment of the essential item and at least two out of six additional signs. These criteria, listed in Table 2, are expected to allow the identification of CAPS patients regardless of the evidence of *NLRP3* mutations, with a sensitivity of 81% and a specificity of 94% [98]. Genetic analyses carried out in patients with suspected CAPS allowed to identify more than 200 *NLRP3* mutations. Most of these are autosomal-dominant or *de novo* mutations, frequently localized in the exon 3 [69]. In addition, several studies described somatic *NLRP3* mosaicisms in patients with early- or late-onset severe clinical features, tested negative for germline mutations [99, 100]. Genotype-phenotype correlation studies divided *NLRP3* variants into three groups, depending on the associated clinical picture, which can be (i) at the ends of the severity spectrum continuum (i.e., FCAS and CINCA); (ii) contiguous CAPS phenotypes, such as FCAS/MWS or MWS/CINCA phenotypes; and (iii) related to polymorphisms or low-penetrance variants. In this regard, initially reported FCAS-associated mutations (including p.L305P, p.L353P, and p.R488K) as well as p.Y570C, p.F309S, or p.F523L which are commonly associated with the severe CINCA phenotype [7, 98, 100] belong to group I. Other mutations instead are common to contiguous CAPS phenotypes: that is the case of p.R260W found in FCAS and CINCA patients, and the case of p.T348M and p.G569R, identified in MWS and CINCA ones [7, 99, 101]. Other mutations, including those affecting the codons p.A439, p.R260, and p.D303, are related to different levels of disease severity, suggesting the involvement of other unknown genetic factors in the development of the CAPS phenotype [101–103]. On the other hand, finding mutations such as p.V198M, p.R488K, and p.Q703K in asymptomatic familial cases, in CAPS patients with heterogeneous phenotypes, and even in healthy controls raised the question of whether these variants might be causative mutations or low-penetrance variants. Indeed, allele frequencies of p.V198M, p.R488K, and p.Q703K were 0.7, 1.4, and 5%, respectively, in healthy Caucasian controls and their clinical significance is still under debate. In particular, the most common low-penetrance variant associated with CAPS milder forms, p.Q703K, was initially described as a neutral polymorphism, subsequently considered a gain-of-function mutation and, according to recent suggestions, redefined as a functional polymorphism rather than a low-penetrance variant [104–106]. More recently, Naselli et al. described the clinical characteristics of 57 patients with the p.Q703K variant: the majority of them displayed a mild CAPS phenotype, characterized by recurrent episodes of urticaria-like rashes and arthralgia. Moreover, monocytes isolated from these patients and stimulated with LPS showed a pattern of cytokine secretion (including IL-1*β*, IL-6, and IL-1 receptor antagonist) similar to that displayed by healthy controls, suggesting that the variant p.Q703K has a limited functional and clinical impact [106].

The *NLRP3* gene encodes an intracellular sensor protein known as cryopyrin, NALP3, or PYPAF1, which is expressed in several cell types, such as monocytes, macrophages, neutrophils, and chondrocytes, and is directly involved in the inflammatory response, being a key component of the multimolecular complex called NLRP3-inflammasome [107–109]. The identification of the NLRP3 inflammasome was a major breakthrough in the field of innate immunity, and research studies following the identification of the CAPS-associated gene led to the characterization of the molecular platform which activates caspase-1, the enzyme required for the proteolytic cleavage and secretion of IL-1*β*. In this context, Agostini et al. demonstrated that this molecular platform includes the sensor protein NLRP3, the adaptor protein ASC, and the cysteine-protease caspase-1 [110], paving the way for the identification of the molecular basis of NLRP3 inflammasome-dependent disorders. Indeed, CAPS-associated mutations, mostly localized in exon 3 encoding for the central nucleotide-binding and oligomerization (NACHT) domain, induce a spontaneous and excessive production of active IL-1*β*. In this regard, monocytes isolated from a p.R260W MWS patient displayed higher IL-1*β* secretion after LPS-mediated NLRP3-inflammasome activation [110]. Similar results were obtained without ATP stimulation in monocytes isolated from CINCA patients carrying the p.N477K, p.D303N, and p.T348M variants [111]. Furthermore, studies on monocytes from patients bearing *NLRP3* mutations associated with CINCA and MWS suggested that an intracellular environment characterized by a deranged redox homeostasis could induce NLRP3-inflammasome activation and IL-1*β* secretion [112]. Interestingly, functional oligomeric NLRP3-inflammasome particles able to amplify extracellular and intracellular caspase-1 proinflammatory activities were detected in the serum of patients with MWS and severe CINCA phenotypes (carrying the p.R260W, p.T348M, p.A439T, and p.D303N mutations) and in CAPS patients with low-grade somatic *NLRP3* mosaicism [113].

### 2.4. Mevalonate Kinase Deficiency (MKD)

MKD encompasses two distinct clinical phenotypes: the most severe mevalonic aciduria (MEVA) and the milder hyperimmunoglobulinemia D syndrome (HIDS). This disease was firstly described in 1984 by van der Meer et al. in six Dutch patients with recurrent attacks of fever and elevated IgD levels [114]. Both MKD phenotypes are caused by the deficient activity of a member of the GHMP (galactokinase, homoserine kinase, mevalonate kinase, and phosphomevalonate kinase) superfamily, i.e., mevalonate kinase (MVK) [5]. MVK catalyzes the conversion of mevalonic acid to 5-phosphomevalonic acid in the second step of the isoprenoid biosynthesis pathway, which supplies the cell with many bioactive molecules, such as isoprenyl groups and sterols [115]. The *MVK* gene, identified as causative for MKD in 1999, is located in the long arm of chromosome 12 at position 24.11: its mutations, inherited in an autosomal recessive manner, lead to decreased MVK activity, as shown by skin fibroblasts from unrelated MKD patients [5]. MKD is a rare condition with an overall higher prevalence in the Netherland and Western Europe. Indeed, the founder mutation p.V377I has been largely reported in many patients from the Netherlands [116, 117]. HIDS is clinically characterized by fever attacks lasting 4-7 days and recurring every 4-6 weeks. Fever is often accompanied by cervical lymphadenopathy, maculopapular rash, mucosal or genital ulcers, and severe abdominal pain with diarrhea and/or vomiting. For HIDS patients, MVK enzyme activity is reduced until 1-10%, while it is reduced to less than 1% in MEVA, which is characterized by serious neurological impairment, failure to thrive, and early death, in addition to HIDS manifestations [118].

Currently, more than 200 *MVK* variants have been related to MKD [69]. Most of them are missense mutations linked to diverse degrees of disease severity [119, 120]. *MVK* deletions, insertions, nonsense mutations, and splicing defects have also been reported. MEVA-associated known mutations are found in regions encoding the active sites of the MVK enzyme, while HIDS-associated ones are found throughout the coding sequence, more often in homozygosity or compound heterozygosity with a second mutation [120]. The most common HIDS-associated variants are p.V377I and p.I268T: the first one is responsible for about 50% of MKD cases and usually detected in compound heterozygosity, rarely in homozygous patients, while p.I268T has been reported in association with both HIDS and MEVA [120]. Other rarer variants (p.A148T, p.P167L, p.N205D, and p.T209A) can cause the HIDS phenotype when a second severe mutation such as p.H20P, p.Y114fs, p.L264F, p.I268T, p.V310M, and p.A334T is present as well [121]. Recently, ter Haar et al. described genotype-phenotype correlations and response to treatment in a large international cohort of 114 MKD patients, observing that the most frequent genotypes are p.V377I/p.I268T and p.V377I/p.V377I, with a frequency of 22% and 12% respectively. Moreover, AA amyloidosis was more often associated with the p.V377I/p.I268T genotype, while patients with variants other than p.V377I showed a more severe musculoskeletal involvement [122].

Isoprenoids are implicated in several biochemical pathways, and one of their functions is the posttranslational modification of small G proteins involved in the inflammatory response: it has been demonstrated that peripheral blood mononuclear cells from patients with HIDS have a limited pool of isoprenoids available and show a decreased Rho GTPase activity: as a consequence, sustained activation of Rac1 (a small GTPase) and increased IL-1*β* production can be observed [123]. Recent data suggest that intracellular oxidative stress and impaired autophagosome degradation stimulate IL-1*β* production [124]. Interestingly, recent discoveries suggest that IL-1*β* release after LPS stimulation of peripheral blood mononuclear cells isolated from HIDS patients (including homozygous and compound heterozygous p.V377I-positive patients) strictly depends on RhoA inactivation, which triggers the pyrin inflammasome [52].

No diagnostic criteria were developed for MKD. However, van der Hilst et al.'s guidelines as well as Eurofever “clinical” classification criteria have been proposed for selecting patients with recurrent fevers worthy of being addressed to *MVK* gene testing [21, 125]. In this respect, it is quite accepted that high serum IgD level is not specific for the diagnosis of HIDS, while intracellular MVK enzyme activity and/or urinary mevalonic acid measured during a fever attack are useful supportive data, especially when genetic analysis is not available or noninformative [21]. Moreover, Steichen et al. suggested that molecular analysis could be avoided when inflammatory attacks arise after the age of 5 years or if febrile flares persist for more than 14 days or if articular pain is absent during inflammatory bouts [126].

## 3. Multifactorial Autoinflammatory Diseases: Working for a Proper Differential Diagnosis

In adult patients, periodic fevers may be sustained by autoinflammatory conditions not directly related to Mendelian inheritance. In particular, adult-onset Still's disease (AOSD), Schnitzler's disease, and periodic fever, aphthous stomatitis, pharyngitis, and cervical adenitis (PFAPA) syndrome should be taken into account for differential diagnosis with mAIDs.

AOSD is a relatively rare condition of unknown origin in which polygenic predisposition, infectious agents, and other environmental factors induce an autoinflammatory systemic response, which gives rise to daily spiking fevers variably associated with evanescent salmon-colored maculopapular rash, arthralgia, myalgia, lymphadenopathy, hepatosplenomegaly with or without increase in aminotransferases, sore throat, mono- or polyserositis, pulmonary infiltrates, and myocarditis. The onset occurs often between 15 and 25 years or between 35 and 45 years. Laboratory assessment discloses marked leukocytosis with neutrophilia, elevated inflammatory markers, and highly increased serum ferritin levels. In particular, ferritin may represent a reliable marker of disease activity. Indeed, serum ferritin levels above 1000 *μ*g/L combined with a glycosylated fraction < 20% has shown to be a highly specific clue for the diagnosis of AOSD [127–129]. In relationship with disease course, AOSD may be alternatively characterized by a unique and self-limited inflammatory bout lasting several weeks to months (monophasic AOSD), but may present a recurrent course with multiple flares (intermittent AOSD) or display a chronic course with persistent polyarthritis and progressive joint destruction (chronic AOSD) [130, 131].

To date, diagnosis of AOSD is based on sets of classification criteria proposed during the last decades, as shown in Table 3 [129, 132]. Those proposed by Yamaguchi et al. are the most widely used and require the preliminary exclusion of infections, cancer, and autoimmune diseases [132]; the criteria proposed by Fautrel et al. are more recent and give a higher weight to ferritin levels and glycosylated ferritin fraction [129]. The Yamaguchi criteria exhibit a sensitivity of 79.2% and specificity of 93.8%; the Fautrel criteria have a sensitivity of 80.6% and specificity of 98.5% [129, 132].

Schnitzler's disease is a very rare inflammatory condition presenting with intermittent fever and urticaria-like skin rashes occurring in subjects with monoclonal gammopathy (mostly of the IgM type) and a considerable increase of inflammatory markers. Arthralgia or arthritis, lymphadenopathy, and liver and/or spleen enlargement are frequent manifestations as well. AA amyloidosis represents a potential long-term consequence of an untreated disease, while monoclonal IgM gammopathy progresses toward an overt lymphoproliferative disorder in about 15-20% of patients [133]. Diagnosis is based on clinical criteria, which have been validated in a cohort of real-life patients, showing a sensitivity for definite and probable diagnosis of 81% and 93%, respectively, with a corresponding specificity of 100% and 97% (see Table 4) [134, 135].

The periodic fever, aphthous stomatitis, pharyngitis, and adenitis (PFAPA) syndrome is an autoinflammatory condition characterized by the clinical features described by the acronym itself. Other various manifestations may occur in addition to cardinal signs, including skin rash, abdominal pain, arthralgia, and conjunctivitis [136–138]. This syndrome is currently the most frequent autoinflammatory cause of noninfective recurrent fever among children in the European population. However, although initially confined to the pediatric world, the PFAPA syndrome has been recently identified as a possible cause of periodic febrile episodes also among adults [139–143]. In this regard, diagnostic criteria specifically tailored on adult patients have been described.  Table 5 illustrates PFAPA diagnostic criteria for both children and adults [144–146]. In relationship with adult patients, the proposed diagnostic criteria identify the PFAPA syndrome among subjects presenting with an increase of inflammatory markers during attacks, symptom-free intervals between inflammatory bouts, and at least one item among erythematous pharyngitis and cervical lymphadenitis during fevers. These criteria should be applied only after having excluded infectious, autoimmune, and neoplastic diseases as well as mAIDs [146].

Behçet's disease (BD), recently classified at the crossroad between autoimmune and polygenic AIDs, may also manifest with otherwise unexplained fever [147, 148]: it is clinically characterized by a classical “triad” of recurrent oral aphthosis, genital ulcers, and uveitis, especially in the form of posterior uveitis and panuveitis. However, inflammation may involve also (i) the gastrointestinal tract mimicking inflammatory bowel diseases, (ii) the vascular tree with multiple venous thrombosis and arterial aneurysms potentially affecting all body sites, (iii) the nervous system manifesting with a wide array of manifestations, most commonly affecting the brainstem and diencephalic regions, and (iv) the skin with erythema nodosum or pseudofolliculitis [149–151]. Diagnosis of BD is based on the fulfillment of international diagnostic criteria and the International Study Group Criteria for BD, which are reported in Table 6 [152, 153].

## 4. Specific Organ Involvement in mAIDs

### 4.1. Constitutional Symptoms

Recurrent fever and asthenia are the main constitutional symptoms in most mAIDs. However, while asthenia is a nonspecific manifestation identified in many systemic disorders, specific inflammatory features associated with recurrent fever may provide valuable clinical information. In particular, FMF attacks generally last between 6 and 72 hours, while TRAPS patients are characterized by episodes lasting 1 to 3 weeks; CAPS and HIDS manifest with fever generally lasting 4-7 days [8, 10, 12, 56, 59]. Of note, also the PFAPA syndrome is characterized by fever episodes resolving in 4-7 days and this disorder should always be taken into account when considering differential diagnosis [136, 137]. Frequency of fever attacks and body temperature may be extremely variable and are less useful for diagnostic purposes.

Systemic reactive AA amyloidosis is the most challenging long-term complication of all mAIDs. Nevertheless, MKD patients are less frequently involved, with a frequency ranging between 3 and 5% [125, 154, 155]. Similarly, amyloidosis is infrequent in patients with FCAS, while nontreated FMF, MWS, and TRAPS may be complicated with systemic amyloidosis in up to 50%, 25%, and 20% of the cases over time, respectively [30].

### 4.2. Skin Manifestations

The skin is frequently involved in mAIDs, and some cutaneous findings may be pathognomonic for a rapid diagnosis. This is the case of erysipelas-like erythema in FMF patients presenting with tender, warm, and swollen erythematous plaques, generally occurring on the distal extremities and triggered by physical activity [10, 12, 156]. Nevertheless, the prevalence of erysipelas-like erythema varies among populations with FMF and is quite uncommon in Arabs and Armenians [157]. Skin biopsy of this lesion might reveal edema with perivascular and interstitial infiltrate consisting of neutrophils and lymphocytes [158, 159].

A centrifugal migratory erythematous rash is the more typical skin manifestation of TRAPS: it is generally accompanied by myalgia due to monocytic fasciitis of the underlying muscles [160, 161]. Although the legs are more frequently involved, the trunk is not spared [13, 68, 156, 161]. A mild perivascular and interstitial infiltrate of mononuclear cells is usually identified at the skin biopsy [156, 160], even though a slight deposition of C3 and C4 in the dermis has also been described [162].

Patients with CAPS present with urticaria-like skin rashes in almost all cases [91]. Indeed, an urticaria-like rash appears among the diagnostic items recently proposed for the clinical assessment of CAPS patients [98]. This rash is typically induced by cold exposure in FCAS, but it may develop at birth or within a few hours after birth in patients with MWS or CINCA/NOMID, disregarding environment temperature. Noteworthy, patients with FCAS are negative to the ice cube test, unlike those affected by cold physical urticaria. Skin biopsy of urticaria-like lesions in CAPS shows a neutrophilic infiltrate of the dermis, with the absence of vasculitis, scarce eosinophils, and sparing of the epidermal layer [156, 163]. Schnitzler's syndrome should be considered for differential diagnosis in patients with monoclonal gammopathy and skin manifestations [134].

Skin lesions are identified in more than two thirds of patients with MKD: they are variable and nonspecific, including maculopapular, morbilliform, erythematous, and purpuric rashes [156, 163, 164]. Oral and/or genital aphthosis can be seen in about half of MKD patients, requiring differential diagnosis with BD, PFAPA syndrome, and FMF [136, 149, 165, 166].

### 4.3. Musculoskeletal Manifestations

Most patients with mAIDs report musculoskeletal manifestations, with FMF and CINCA/NOMID being the most typically involved. Nevertheless, patients with MKD and TRAPS may refer joint and muscle symptoms as well [8, 10, 12, 56, 59].

Acute monoarthritis may be the sole disease manifestation in up to 15% of FMF patients [167–169]. Nonetheless, monoarthritis is less frequent when patients are genetically diagnosed, but do not fulfill clinical diagnostic criteria for FMF [169]. In these cases, symmetrical acute arthritis of large joints of the lower limbs is the most frequent finding. Alternatively, subacute or chronic articular involvement may be identified [170, 171]. Although the knee and hip are more frequently involved, FMF-related arthritis may affect all articular sites, including the elbow, sternoclavicular joints, and metacarpophalangeal joints [172, 173]. Also sacroiliitis may be identified in FMF and seems to be closely related to the M694V *MEFV* mutation that has been suggested as a susceptibility factor for enthesitis-related arthritis [174–178]. While myositis is uncommon in FMF, myalgia is reported in up to 25% of the patients and may be classified as spontaneous, induced by exercise, or protracted febrile myalgia [179, 180]. Specifically, protracted febrile myalgia is a debilitating manifestation often accompanied by other symptoms such as abdominal pain, diarrhea, arthritis/arthralgia, and transient vasculitic lesions mimicking the Henoch-Schönlein purpura. Electromyography shows only nonspecific changes, while muscle enzymes are generally within the normal range [181]. There is some evidence that musculoskeletal symptoms may occur more frequently in patients with late-onset disease than in those displaying an early onset [182].

Articular involvement in CAPS affects approximately 60% of patients and ranges from arthralgia with transient joint swelling during flares to chronic disabling joint disease [91, 95]. Severe joint manifestations including chronic arthritides with structural changes and bony overgrowth are described in patients with CINCA/NOMID and involve especially the knees. These articular affections bring about severe functional impairment since early adulthood [91, 183–185].

With regard to TRAPS, myalgia with monocytic fasciitis in the absence of direct inflammatory involvement of the muscle is a characteristic feature of the disease. Therefore, serum levels of muscle enzymes are usually normal [160, 161]. Myalgia is generally associated with warm and tender patches of the overlying skin, which are recognized as typical features of TRAPS [61, 70, 71, 160, 161]. Regarding joint involvement, TRAPS patients often suffer from arthralgia, while arthritis is an unusual finding that eventually affects large joints in a nonerosive fashion [186]. Despite this, the involvement of small joints and sacroiliitis have been anecdotally reported in subjects carrying low-penetrance mutations [187].

Polyarthralgia is observed in approximately 80% of MKD patients, while nonerosive arthritis of large joints and myalgia are reported in about half of the cases. Musculoskeletal manifestations may be severe and disabling in about 7-8% of patients, who might develop flexion contractures, bone erosions, and even deformities. Articular symptoms may persist also during symptom-free intervals of MKD patients [155].

### 4.4. Serosal Involvement in mAIDs

Recurrent serosal acute inflammation may be a presenting peculiar manifestation of mAIDs. In particular, recurrent pleuritis and peritonitis represent typical features of FMF and they are counted among the major items in the clinical diagnostic criteria [28, 55, 65]. Similarly, chest pain and abdominal pain, which are possibly due to pericarditis, pleuritis, or peritonitis, appear among the pediatric FMF criteria [56].

Notably, although the incidence of pericarditis has been reported to be quite low in FMF [188, 189], echocardiography may identify a subclinical involvement in up to 27% of patients, especially among subjects with a longer disease duration [190].

Recurrent acute pericarditis has been largely described in patients with TRAPS. Of note, recurrent acute pericarditis may be the sole clinical manifestation in subjects carrying low-penetrance mutations [66, 74, 160, 191, 192]. Therefore, clinical clues have been proposed to identify among patients with idiopathic recurrent acute pericarditis those who potentially might carry *TNFRSF1A* gene mutations: a positive family history for either pericarditis or recurrent fevers, a high rate of recurrences beyond the first year of disease, colchicine resistance, and the need for immunosuppressants to achieve control of the disease should induce a clinical suspicion of TRAPS [64, 67].

Although not included in the clinical diagnostic criteria, pleuropericarditis is frequently identified also in patients with AOSD [193].

### 4.5. Gastrointestinal Manifestations

Although gastrointestinal symptoms may be found in almost all mAIDs, abdominal pain with or without diarrhea and vomiting are more frequently found in FMF and HIDS [125, 194]. Abdominal pain in FMF is closely related to aseptic recurrent peritonitis and usually represents a prevailing clinical manifestation. For this reason, abdominal pain (with or without peritonitis) appears in all clinical FMF criteria [55, 56] as well as in the more recent Eurofever classification criteria [21]. Although less useful for diagnostic purposes, other gastrointestinal manifestations have been often reported in FMF patients [195]. Noteworthy, inflammatory bowel diseases seem to be more frequent and severe in patients with FMF [196] and, conversely, the rate of *MEFV* mutations has been found higher in subjects diagnosed with inflammatory bowel diseases [197].

Abdominal pain, vomiting, and/or diarrhea along with oral aphthosis are almost constant in HIDS patients during fever attacks. Sterile peritonitis mimicking acute appendicitis can lead to surgical interventions, peritoneal adhesions, and intestinal obstruction [125]. Based on the higher frequency of gastrointestinal affections, the occurrence of diarrhea during fever attacks has been included in the Eurofever classification criteria [21].

Notably, abdominal pain and intestinal inflammation may be also manifestations of BD, which should be ruled out during the diagnostic process [152, 153]. Similarly, the PFAPA syndrome may be associated with abdominal pain and various intestinal disorders [198].

### 4.6. Ocular Manifestations

No specific ocular involvement is reported in FMF. However, fundus abnormalities resembling colloid bodies in the Bruch membrane have been described on routine funduscopic examination during FMF fever attacks [199, 200]. In addition, increased choroidal thickness has been identified inconsistently during attacks [201]. Anterior, posterior, and intermediate uveitis have been occasionally described in FMF patients [202]. Conversely, FMF could represent a predisposing factor for the development of keratoconus, especially in patients carrying homozygous mutations [203].

In TRAPS patients, the eye is most commonly affected with periorbital edema or pain and conjunctivitis [61]. Periorbital edema is more common in pediatric patients as well as in adults with an early disease onset. On the contrary, eye manifestations are less frequently encountered in patients carrying the R92Q low-penetrance *TNFRSF1A* mutation [60]. Nevertheless, bilateral panuveitis has been recently detected in a child with TRAPS carrying the R92Q variant [204].

Retinitis pigmentosa and cataract may be recognized in patients with MKD, and in particular retinitis pigmentosa may occasionally represent the presenting feature of the disease [205–207].

Optic disc changes including papilledema and optic atrophy are the most common ocular findings in MWS and CINCA/NOMID [91]. In this regard, the gradual loss of nerve fibers owing to optic disc alterations explains gradual visual loss in such patients [208]. Of note, retinal vasculitis, focal choroiditis, vitritis, band keratopathy, nummular stromal infiltration, corneal vascularization, and cataract are rare manifestations occasionally encountered in CINCA/NOMID [209, 210], while midstromal changes, episcleritis, keratitis, and uveitis have been reported in MWS [211, 212]. Lastly, eye involvement is mainly represented by conjunctivitis in FCAS patients. Keratitis with bilateral corneal scars have been reported only in severe cases [213].

Ocular inflammatory involvement should also pave the attention to BD, which may manifest with recurrent, unilateral or bilateral panuveitis, posterior uveitis, and/or retinal vasculitis; less frequently, anterior uveitis can occur [152, 153].

Ophthalmologists should be aware of the possibility of severe ocular manifestations related to mAIDs, and that ocular inflammatory signs might even occur in the context of mild phenotypes [214].

### 4.7. Neurological Features

Among mAIDs, neurological manifestations are more closely related to severe CAPS phenotypes. Indeed, while headache and drowsiness are the only neurological symptoms of FCAS during inflammatory bouts, MWS is often complicated by sensorineural deafness and progressive visual loss due to the atrophy of the optic nerve over time [8, 12, 56, 59, 91]. The nervous system is severely affected in CINCA/NOMID: suggestive clinical signs include chronic aseptic meningitis, increased intracranial pressure, cerebral atrophy, hydrocephalus or ventriculomegaly, sensorineural hearing loss, and chronic papilledema [91]. Sterile meningeal inflammation, which represents the major cause of morbidity and mortality in such patients, is found in almost all cases and contributes to the onset of mental retardation and seizures in at least half of CINCA patients. Hydrocephalus and ventriculomegaly are often present at birth or even observed in utero, possibly constituting the initial presentation of the disease. Less common neurological signs include diplegia, hypotonia, or transient episodes of hemiplegia [215–218].

Aseptic meningitis and seizures are neurologic manifestations infrequently occurring in FMF patients [219, 220]. However, myalgia is the most common neurological symptom in FMF. Headache is very frequent in TRAPS and MKD patients. In addition, psychomotor retardation, progressive cerebellar ataxia, hypotonia, and developmental delay may be identified in patients with MEVA [8, 12, 56, 59, 116, 221].

Among polygenic AIDs, BD might start with severe signs of neurological involvement, consisting of vascular affections with variable severity inducing stroke-related manifestations [222–224].

### 4.8. Visceral Involvement

Lymphadenopathy is a frequent finding in different inflammatory disorders, including mAIDs. Noteworthy, the enlargement of the cervical lymph node represents a diagnostic clue for patients with the PFAPA syndrome and should be taken into account for patients presenting with laterocervical lymphadenopathy solely during fever attacks. Cervical lymphadenitis is part of diagnostic criteria drafted for both PFAPA children and adults [136–138, 144–146].

Lymphadenopathy and/or splenomegaly also appear among minor items of the Yamaguchi criteria for the clinical diagnosis of AOSD [132]. Regarding splenomegaly, no specific diagnostic role has been currently recognized in mAIDs. However, spleen enlargement may be found in more than 60% of patients with MKD and in up to 12.6% of patients with FMF [225, 226].

A persistent systemic inflammation may impact on liver function, causing nonamyloid chronic hepatitis and even cryptogenic cirrhosis in some patients with FMF [26, 227]. Conversely, AOSD may show elevated liver enzymes during inflammatory episodes, and increased serum aminotransferases have been included in the diagnostic criteria by Yamaguchi et al. for the clinical diagnosis of AOSD [132].

No studies are available to indicate endocrine abnormalities in patients with mAIDs, although growth hormone deficiency has been reported in one CINCA patient with a severe skeletal picture who also displayed a substantial response to recombinant human growth hormone therapy [228].

Lung involvement is infrequent in patients with mAIDs. Actually, pleuritis is the most common pulmonary manifestation of FMF [8, 12, 56, 59, 65, 229]. Conversely, regarding atypical manifestations, recurrent pulmonary atelectasis and isolated pulmonary vasculitis have been rarely reported in FMF [230, 231]. Lung amyloidosis is a further rare complication of nontreated FMF, characterized by clinical and radiologic findings that may mimic chronic interstitial lung disease. Nevertheless, lung amyloidosis generally follows amyloid nephropathy [229]. Pleuritis may be found also in patients with TRAPS [65].

## 5. Conclusive Remarks

The diagnostic assessment of adult patients referred for recurrent fevers can be highly challenging. After the exclusion of infectious, immune-mediated, oncologic, and blood diseases, the suspicion should be also directed to the field of autoinflammation and both monogenic or multifactorial (or polygenic) AIDs should be taken into accurate consideration. The spectrum of mAIDs is evolving rapidly, following the availability of more sophisticated and cost-effective genetic sequencing techniques. Over the past decades, many new clinical entities have been disclosed and classified among mAIDs, sometimes at the crossroad of autoinflammation, immune deficiencies, immune deregulation, and autoimmunity; on the other hand, well-known multifactorial diseases, i.e., nonhereditary collagen-like diseases, idiopathic inflammatory disorders, and metabolic diseases, have been reclassified among AIDs in the light of a deeper insight about their pathogenetic mechanisms which included autoinflammation [232, 233].

Although mAIDs are rare diseases, a wider awareness of these disorders among physicians from different specialties is bringing about a sizeable increase in the identification of patients, even with noncanonical presentation which varies from atypical unusual or nuanced symptoms to macrophage activation syndrome [234, 235]. The first step for the diagnosis of mAIDs is to review carefully all details of fever episodes and response to treatment, since examination between attacks may be unrevealing, and tailor the potentially enormous laboratory evaluation to a few tests focusing on the most likely diagnoses. For instance, a favourable response to colchicine prophylaxis is a major diagnostic clue to the diagnosis of FMF [236], whereas constitutive activation of the IL-1 secreting platform is the backstory of CAPS, which dramatically responds to IL-1 antagonists [237]. The discovery of a causative link between autoinflammation and IL-1 release has improved our understanding of the intimate mechanisms of innate immunity, and have likewise led to the identification of extraordinary treatments for many of these disorders [238–240]. Moreover, during the last years, different diagnostic scores have been proposed to improve the identification of people with mAIDs or optimize their access to genetic testing, and also different scores to measure chronic damage have been developed [241, 242]. The increasing reports of adults with mAIDs have helped clarify the connections between innate immunity and environment, although a specific diagnosis still requires the integration of different clinical and laboratory data, family history, ethnicity, and focused genetic analysis.

**Figure 2:**
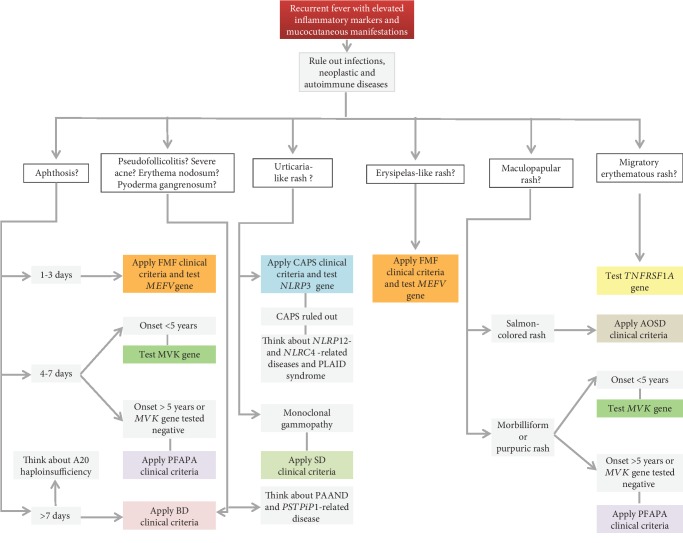
Diagnostic flow chart for main autoinflammatory diseases displaying recurrent fevers and mucocutaneous manifestations. List of abbreviations: *FMF*—familial Mediterranean fever; *PFAPA*—periodic fever, aphthous stomatitis, pharyngitis, and cervical adenitis; *BD*—Behçet's disease; *CAPS*—cryopyrin-associated periodic syndrome; *SD*—Schnitzler's disease; *AOSD*—adult-onset Still's disease; *NLRP12*—nucleotide-binding domain and leucine-rich repeat-containing protein 12; *NLRC4*—nucleotide-binding domain, leucine-rich repeat, and caspase recruitment domain-containing 4; *PLAID*—phospholipase C gamma 2-associated antibody deficiency and immune dysregulation; *PAAND*—pyrin-associated autoinflammation with neutrophilic dermatosis; *PSTPiP1*—proline-serine-threonine phosphatase interacting protein 1.

**Figure 3:**
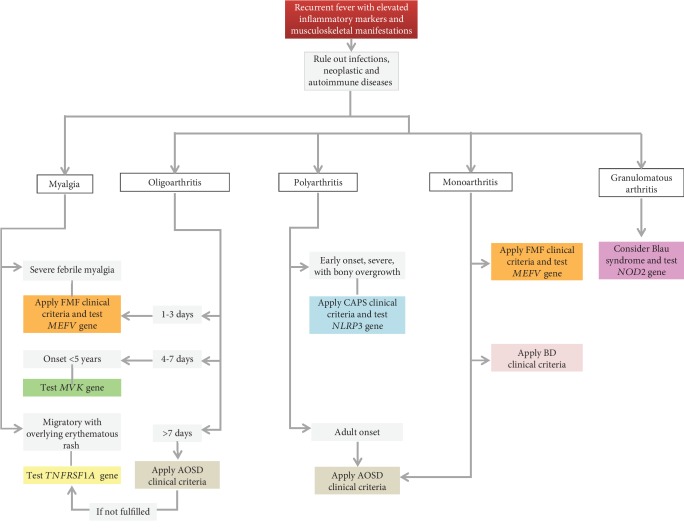
Diagnostic flow chart for main autoinflammatory diseases displaying recurrent fevers and musculoskeletal manifestations. List of abbreviations: *FMF*—familial Mediterranean fever; *AOSD*—adult-onset Still's disease; *BD*—Behçet's disease; *CAPS*—cryopyrin-associated periodic syndrome.

**Figure 4:**
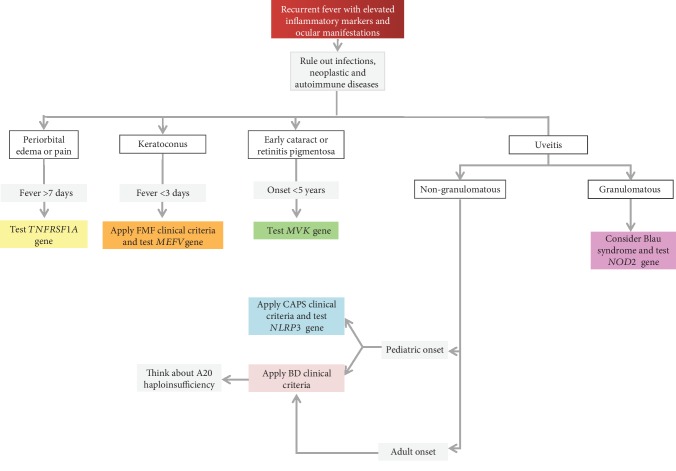
Diagnostic flow chart for main autoinflammatory diseases displaying recurrent fevers and ocular manifestations. List of abbreviations: *FMF*—familial Mediterranean fever; *BD*—Behçet's disease; *CAPS*—cryopyrin-associated periodic syndrome.

**Figure 5:**
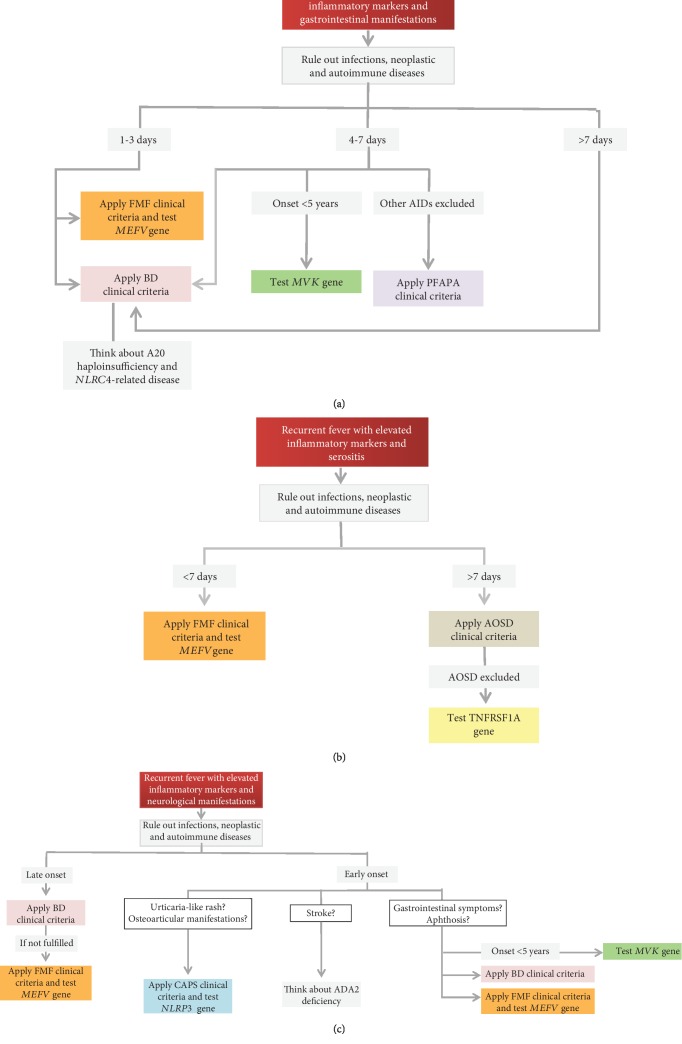
Diagnostic flow chart for main autoinflammatory diseases displaying recurrent fevers and gastrointestinal (a), serosal (b), and neurological (c) manifestations. List of abbreviations: *FMF*—familial Mediterranean fever; *PFAPA*—periodic fever, aphthous stomatitis, pharyngitis, and cervical adenitis; *BD*—Behçet's disease; *CAPS*—cryopyrin-associated periodic syndrome; *AOSD*—adult-onset Still's disease; *NLRC4*—nucleotide-binding domain, leucine-rich repeat, and caspase recruitment domain-containing 4; *ADA2*—adenosine deaminase 2.

**Figure 6:**
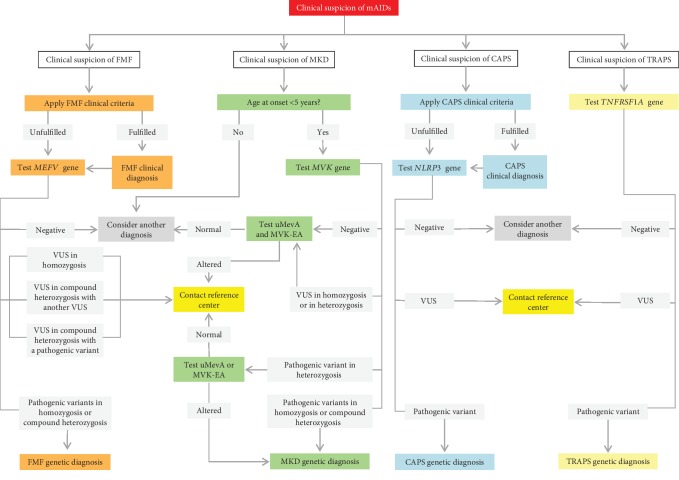
Genetic evaluation in patients with a suspicion of FMF, MKD, CAPS, and TRAPS. List of abbreviations: *FMF*—familial Mediterranean fever; *MKD*—mevalonate kinase deficiency; *CAPS*—cryopyrin-associated periodic syndrome; TRAPS—tumor necrosis factor receptor-associated periodic syndrome; *uMevA*—urinary mevalonic acid during fever; *MVK-EA*—mevalonate kinase enzyme activity; *VUS*—variant of unknown significance.

In this review, we have summarized the clinical features shared by the most common monogenic and multifactorial AIDs, with the intent to offer a practical guide to the clinician involved in the management of patients with a history of recurrent fevers (Figures 2–5). With the same purpose, we provided an overview of genetic variants associated with hereditary recurrent febrile syndromes and their pathogenetic significance (Table 7) and we suggested an empirical flow chart for genetic diagnosis (Figure 6). In closing, the clinical judgement of the rheumatologist, in close collaboration with the geneticist and the multidisciplinary équipe experienced in autoinflammation, remains the architrave to unravel the multifaceted complexity of AIDs in real-life scenarios.

## Figures and Tables

**Table 1:** Summary of the main genetic and clinical features of FMF, TRAPS, CAPS, and MKD.

Disease	Gene locus	Inheritance	Protein	Main clinical features
FMF	*MEFV* 16p13.3	AR	Pyrin	Fever, serositis, arthritis generally affecting large joints, erysipelas-like rash, and systemic AA amyloidosis in untreated patients

TRAPS	*TNFRSF1A* 12p13	AD	Tumor necrosis factor receptor 1	Fever, migrating erythematous skin rash, muscle pain due to monocytic fasciitis, periorbital edema, arthralgia or arthritis, serosal involvement, and systemic AA amyloidosis in untreated patients

FCAS	*NLRP3* 1q44	AD	Cryopyrin	Fever, cold-induced urticaria-like rash, conjunctivitis, and arthralgia
MWS				Fever, urticaria-like rash, conjunctivitis, arthralgia, neurosensorial deafness, and risk of amyloidosis
CINCA				Fever, urticaria-like rash, uveitis, papilledema, deforming arthritis mainly involving large joints, chronic aseptic meningopathy, neurosensorial deafness, and risk of amyloidosis

HIDS	*MVK* 12q24	AR	Mevalonate kinase	Fever, polymorphous rash, arthralgia, abdominal pain, diarrhea, lymph node enlargement, splenomegaly, and aphthosis
MEVA				Psychomotor retardation, growth delay, progressive cerebellar ataxia, dysmorphisms, and vision deficits in addition to HIDS features

List of abbreviations: AD—autosomal dominant; AR—autosomal recessive; CINCA—chronic infantile neurologic cutaneous and articular syndrome; FCAS—familial cold autoinflammatory syndrome; FMF—familial Mediterranean fever; HIDS—hyperimmunoglobulinemia D syndrome; *MEFV*—Mediterranean fever; MEVA—mevalonic aciduria; MKD—mevalonate kinase deficiency; *MVK*—mevalonate kinase; MWS—Muckle-Wells syndrome; *NLRP3*—NACHT, LRR, and PYD domain-containing protein 3; *TNFRSF1A*—tumor necrosis factor receptor super family 1A; TRAPS—tumor necrosis factor-associated periodic syndrome.

**Table 2:** Diagnostic criteria for patients with suspected cryopyrin-associated periodic syndrome (CAPS); adapted from Kuemmerle-Deschner et al. [98].

Mandatory item
Increase of inflammatory markers (C-reactive protein and/or serum amyloid A)

Additional items
(i) Urticaria-like rash (ii) Cold-triggered episodes (iii) Sensorineural hearing loss (iv) Musculoskeletal symptoms (v) Chronic aseptic meningitis (vi) Skeletal abnormalities

At least 2 additional items are requested

**Table 3:** Clinical criteria for the diagnosis of adult onset Still's disease (AOSD); adapted from Yamaguchi et al. [132] and Fautrel et al. [129]. Infections, malignancies, and other rheumatologic diseases represent exclusion criteria.

*Yamaguchi criteria*	*Fautrel criteria*
Major criteria	Major criteria
(i) Fever > 39°C, intermittent, lasting at least one week	(i) Spiking fever ≥ 39°C
(ii) Arthralgia for at least 2 weeks	(ii) Arthralgia
(iii) Typical skin rash	(iii) Transient erythema
(iv) White blood cells ≥ 10, 000/mm^3^ with granulocytes > 80%	(iv) Pharyngitis
	(v) Blood polymorphonuclear leukocytes ≥ 80%
	(vi) Glycosylated ferritin ≤ 20%

Minor criteria	Minor criteria
(i) Sore throat	(i) Maculopapular skin rash
(ii) Lymphadenopathy and/or splenomegaly	(ii) White blood cells ≥ 10, 000/mm^3^
(iii) Abnormal liver function tests	
(iv) Negative antinuclear antibodies and rheumatoid factor	

At least 5 items, including 2 major criteria, are requested	4 major criteria or 3 major+2 minor items are requested

**Table 4:** Clinical criteria for the diagnosis of Schnitzler's disease [134]. A definite diagnosis is justified by the fulfillment of the 2 mandatory criteria and at least 2 or 3 minor criteria in patients with IgM or IgG monoclonal gammopathy, respectively. Diagnosis is probable when the 2 mandatory criteria are fulfilled and at least 1 or 2 minor criteria in patients with IgM or IgG monoclonal gammopathy, respectively, are present.

Mandatory criteria
(i) Chronic urticaria-like skin rash (ii) IgM or IgG monoclonal gammopathy

Minor criteria
(i) Unexplained recurrent fever > 38°C (ii) Abnormal bone remodeling assessed by bone scintigraphy or magnetic resonance imaging with abnormal bone alkaline phosphatase level (iii) Neutrophilic dermal infiltrate at skin biopsy with neither fibrinoid necrosis nor significant dermal edema (iv) Leukocytosis (neutrophils > 10, 000/mm^3^) and/or elevated C-reactive protein (>30 mg/l)

**Table 5:** Clinical criteria for the diagnosis of periodic fever, aphthous stomatitis, pharyngitis, and cervical adenitis (PFAPA) syndrome in children and adults; adapted from Thomas et al. [145] and Cantarini et al. [146], respectively. Infections, malignancies, autoimmune and other autoinflammatory diseases should be ruled out before the application of criteria in adult patients.

*Diagnostic criteria for children*	*Diagnostic criteria for adults*
(1) Regularly recurring fevers with early age of onset (<5 years of age), occurring in the absence of upper respiratory tract infections (2) At least one among the following symptoms: (i) Aphthous stomatitis (ii) Cervical lymphadenitis (iii) Pharyngitis (3) Exclusion of cyclic neutropenia (4) Asymptomatic interval between episodes (5) Normal growth and development	(1) Recurrent fever accompanied by (i) Erythematous pharyngitis *and/or* (ii) Cervical lymphadenitis (2) Increased inflammatory markers during febrile attacks (3) Asymptomatic interval between episodes
All the criteria are requested	All the criteria are requested

**Table 6:** Criteria for the diagnosis of Behçet's disease (BD): International Study Group Criteria (ISGC) [152] and International Criteria for BD (ICBD) [153].

*ISGC*	*ICBD*	
Mandatory item	Items	Score
(i) Recurrent oral aphthosis (at least 3 episodes per year)	(i) Oral aphthosis	2
Additional items	(ii) Genital aphthosis	2
(i) Recurrent genital ulcers (ii) Ocular or retinal inflammatory manifestations (iii) Skin lesions (papulopustular lesions, erythema nodosum) (iv) Positive pathergy reaction	(iii) Ocular inflammatory lesions	2
	(iv) Skin lesions	1
	(v) Neurological involvement	1
	(vi) Vascular manifestations	1
	(vii) Positive pathergy test	1
At least 2 additional items are requested	*Cut-off*	≥4

**Table 7:** Genetic variants associated with hereditary recurrent febrile syndromes and interpretations of their significance (from ClinVar database).

Disease	Gene—locus (NM)	Location	Sequence variant (protein variant)	SNP no.	Clinical significance
Familial Mediterranean fever (FMF, #249100)	*MEFV*—16p13.3 (NM_000243.2)	*Exon 1*	c.97G>T (p.Val33Leu)	rs11466016	US
		*Exon 2*	c.289C>T (p.Gln97Ter)	rs138498376	US
		*Exon 2*	c.277G>C (p.Glu93Gln)	rs747515115	US
		*Exon 2*	c.329T>C (p.Leu110Pro)	rs11466018	US
		*Exon 2*	c.343C>A (p.Pro115Thr)	rs147557169	US
		*Exon 2*	c.442G>C (p.Glu148Gln)	rs3743930	P/US/LB
		*Exon 2*	c.443A>T (p.Glu148Val)	rs104895076	P/US
		*Exon 2*	c.501G>C (p.Glu167Asp)	rs104895079	P
		*Exon 2*	c.605G>A (p.Arg202Gln)	rs224222	LB/B
		*Exon 2*	c.611G>A (p.Arg204His)	rs775663363	US
		*Exon 2*	c.688G>A (p.Glu230Lys)	rs104895080	US
		*Exon 2*	c.800C>T (p.Thr267Ile)	rs104895081	P
		*Exon 2*	c.910G>A (p.Gly304Arg)	rs75977701	US/LB
		*Exon 3*	c.941G>A (p.Arg314His)	rs104895204	US
		*Exon 3*	c.986G>A (p.Arg329His)	rs104895112	US
		*Exon 3*	c.1016C>T (p.Ser339Phe)	rs104895157	US
		*Exon 3*	c.1043G>A (p.Arg348His)	rs104895198	US
		*Exon 3*	c.1105C>T (p.Pro369Ser)	rs11466023	P/LP/US
		*Exon 3*	c.1222C>T (p.Arg408Trp)	rs758868622	US
		*Exon 3*	c.1223G>A (p.Arg408Gln)	rs11466024	P/US/B
		*Exon 3*	c.1223G>T (p.Arg408Leu)	rs11466024	US
		*Exon 3*	c.1318C>G (p.Gln440Glu)	rs11466026	US/B
		*Exon 5*	c.1370C>T (p.Ala457Val)	rs104895151	US
		*Exon 5*	c.1406T>C (p.Val469Ala)	rs778686119	US
		*Exon 5*	c.1437C>G (p.Phe479Leu)	rs104895083	P
		*Exon 5*	c.1459G>C (p.Val487Leu)	rs104895100	LB
		*Exon 5*	c.1508C>G (p.Ser503Cys)	rs190705322	US
		*Exon 5*	c.1513G>T (p.Asp505Tyr)	rs150730718	US
		*Exon 8*	c.1730C>A (p.Thr577Asn)	rs1057516210	P
		*Exon 8*	c.1736G>A (p.Arg579His)	rs574055513	US
		*Exon 9*	c.1772T>C (p.Ile591Thr)	rs11466045	P/US/LB
		*Exon 10*	c.1894G>A (p.Gly632Ser)	rs104895128	LP
		*Exon 10*	c.1898C>T (p.Pro633Leu)	rs976279218	US
		*Exon 10*	c.1958G>A (p.Arg653His)	rs104895085	P
		*Exon 10*	c.2040G>A (p.Met680Ile)	rs28940580	P
		*Exon 10*	c.2040G>C (p.Met680Ile)	rs28940580	P
		*Exon 10*	c.2064C>G (p.Tyr688Ter)	rs104895098	P
		*Exon 10*	c.2076_2078delAAT (p.Ile692del)	rs104895093	P
		*Exon 10*	c.2080A>G (p.Met694Val)	rs61752717	P
		*Exon 10*	c.2081_2083delTGA (p.Met694del)	rs104895091	P
		*Exon 10*	c.2082G>A (p.Met694Ile)	rs28940578	P
		*Exon 10*	c.2084A>G (p.Lys695Arg)	rs104895094	P/LP/LB
		*Exon 10*	c.2177T>C (p.Val726Ala)	rs28940579	P
		*Exon 10*	c.2230G>T (p.Ala744Ser)	rs61732874	P/LP
		*Exon 10*	c.2282G>A (p.Arg761His)	rs104895097	P/LP
		*Exon 10*	c.2330_2331del (p.(Gly777Alafs^∗^4))	rs753946287	US

Hyperimmunoglobulinemia D syndrome (HIDS, #610377)	*MVK*—12q24 (NM_052845.3)	*Exon 2*	c.16_34del (p.(Leu6Glyfs^∗^16))	rs104895334	P
		*Exon 2*	c.56G>A (p.Arg19His)	rs10774775	B
		*Exon 2*	c.59A>C (p.His20Pro)	rs104895295	P
		*Exon 3*	c.72dup (p.(Gly25Trpfs^∗^55))	rs104895322	P
		*Exon 3*	c.155G>A (p.Ser52Asn)	rs7957619	LB/B
		*Exon 4*	c.238G>A (p.Val80Ile)	rs76914224	US/LB
		*Exon 4*	c.302G>A (p.Cys101Tyr)	rs886048931	US
		*Exon 4*	c.317G>A (p.Arg106His)	rs778337320	US
		*Exon 4*	c.331G>A (p.Ala111Thr)	rs371257609	US
		*Exon 4*	c.346T>C (p.Tyr116His)	rs104895382	P
		*Exon 5*	c.417dup (p.(Gly140Argfs^∗^47))	rs104895373	P
		*Exon 5*	c.421dup (p.(Ala141Glyfs^∗^46))	rs104895323	P
		*Exon 5*	c.442G>A (p.Ala148Thr)	rs104895298	P
		*Exon 5*	c.494C>T (p.Pro165Leu)	rs121917790	P
		*Exon 5*	c.500C>T (p.Pro167Leu)	rs104895300	P/LP
		*Exon 6*	c.564G>A (p.Trp188Ter)	rs104895311	P
		*Exon 6*	c.598C>T (p.Pro200Ser)	rs886048932	US
		*Exon 6*	c.604G>A (p.Gly202Arg)	rs104895301	P
		*Exon 6*	c.608T>C (p.Val203Ala)	rs104895332	P
		*Exon 8*	c.709A>T (p.Thr237Ser)	rs104895366	P
		*Exon 9*	c.803T>C (p.Ile268Thr)	rs104895304	P
		*Exon 9*	c.857C>T (p.Pro286Leu)	rs104895379	US
		*Exon 10*	c.904C>T (p.Gln302Ter)	rs886048933	LP/US
		*Exon 10*	c.928G>A (p.Val310Met)	rs104895319	P
		*Exon 10*	c.1000G>A (p.Ala334Thr)	rs104895317	P
		*Exon 11*	c.1129G>A (p.Val377Ile)	rs28934897	P
		*Exon 11*	c.1156G>A (p.Asp386Asn)	rs104895380	LB
		*Exon 11*	c.1162C>T (p.Arg388Ter)	rs104895360	P
		*Exon 11*	c.1163G>A (p.Arg388Gln)	rs886048934	US

Tumor necrosis factor-associated periodic syndrome (TRAPS, #142680)	*TNFRSF1A*—12p13 (NM_001065.3)	*Exon 2*	c.92T>G (p.Val31Gly)	rs763940329	US
		*Exon 2*	c.123T>G (p.Asp41Glu)	rs104895271	LP
		*Exon 2*	c.175T>C (p.Cys59Arg)	rs104895217	P
		*Exon 2*	c.176G>C (p.Cys59Ser)	rs104895223	P
		*Exon 2*	c.176G>A (p.Cys59Tyr)	rs104895223	US
		*Exon 2*	c.184T>G (p.Cys62Gly)	rs104895225	P
		*Exon 2*	c.185G>A (p.Cys62Tyr)	rs104895218	P
		*Exon 3*	c.211_213delGAC (p.Asp71del)	rs104895246	P
		*Exon 3*	c.224C>T (p.Pro75Leu)	rs4149637	LB/B
		*Exon 3*	c.236C>T (p.Thr79Met)	rs104895219	P
		*Exon 3*	c.242G>T (p.Cys81Phe)	rs104895220	P
		*Exon 3*	c.265T>C (p.Phe89Leu)	rs104895245	LP
		*Exon 3*	c.282C>G (p.Asn94Lys)	rs876661014	LP
		*Exon 3*	c.287T>C (p.Leu96Pro)	rs104895235	US
		*Exon 3*	c.295T>A (p.Cys99Ser)	rs104895228	P
		*Exon 3*	c.295T>C (p.Cys99Arg)	rs104895228	P
		*Exon 3*	c.305G>C (p.Cys102Ser)	—	LP
		*Exon 3*	c.317G>A (p.Arg106Gln)	rs876661031	LP
		*Exon 4*	c.334G>A (p.Val112Met)	rs201753543	US
		*Exon 4*	c.349T>C (p.Cys117Arg)	rs104895221	P
		*Exon 4*	c.350G>A (p.Cys117Tyr)	rs104895222	P
		*Exon 4*	c.362G>A (p.Arg121Gln)	rs4149584	P/US/LB/B
		*Exon 4*	c.362G>C (p.Arg121Pro)	rs4149584	P
		*Exon 4*	c.370G>A (p.Val124Met)	rs104895278	US
		*Exon 4*	c.434A>G (p.Asn145Ser)	rs104895288	US
		*Exon 5*	c.532G>A (p.Glu178Lys)	rs538872981	US
		*Exon 6*	c.596T>C (p.Ile199Thr)	rs104895247	P/US
		*Exon 9*	c.806C>G (p.Pro269Arg)	rs876661237	US
		*Exon 9*	c.823C>T (p.Pro275Ser)	rs758118907	US
		*Exon 9*	c.935G>A (p.Arg312Lys)	—	P/LB/B
		*Exon 9*	c.959G>A (p.Gly320Glu)	rs1057524143	LB
		*Exon 9*	c.988G>A (p.Ala330Thr)	rs200029309	US/LB
		*Exon 10*	c.1159C>T (p.Arg387Trp)	—	US
		*Exon 10*	c.1234C>G (p.Pro412Ala)	rs876661181	US
		*Exon 10*	c.1328G>T (p.Gly443Val)	rs201062001	US
		*Exon 10*	c.1356T>A (p.Ser452Arg)	rs886049750	US

Cryopyrin-associated periodic syndrome: familial cold autoinflammatory syndrome (FCAS, #120100), Muckle-Wells syndrome (MWS, #191900), chronic infantile neurological cutaneous articular syndrome (CINCA, #607115)	*NLRP3*—1q44 (NM_001243133.1)	*Exon 1*	c.61G>C (p.Asp21His)	rs200154873	P
		*Exon 1*	c.82C>T (p.His28Tyr)	rs763551829	US
		*Exon 1*	c.152A>G (p.His51Arg)	rs367663649	US
		*Exon 1*	c.178G>A (p.Asp60Asn)	rs1131691891	US
		*Exon 1*	c.200C>G (p.Ala67Gly)	rs763252989	US
		*Exon 1*	c.209T>C (p.Met70Thr)	rs147559626	LB
		*Exon 1*	c.214G>A (p.Val72Met)	rs117287351	LB
		*Exon 1*	c.230C>A (p.Ala77Glu)	rs200288250	US
		*Exon 1*	c.230C>T (p.Ala77Val)	rs200288250	US
		*Exon 2*	c.298C>T (p.Arg100Cys)	rs375013904	US
		*Exon 2*	c.299G>A (p.Arg100His)	rs201887896	US
		*Exon 2*	c.392A>G (p.Lys131Arg)	rs188623199	US
		*Exon 3*	c.410G>A (p.Arg137His)	rs138946894	US
		*Exon 3*	c.494A>G (p.Asn165Ser)	rs199475733	US/LB
		*Exon 3*	c.584C>T (p.Thr195Met)	rs76291085	US
		*Exon 3*	c.592G>A (p.Val198Met)	rs121908147	P/US/LB/B
		*Exon 3*	c.634G>A (p.Asp212Asn)	rs372038150	US
		*Exon 3*	c.644A>G (p.His215Arg)	rs150396172	US
		*Exon 3*	c.674C>T (p.Ala225Val)	rs180177493	US
		*Exon 3*	c.749A>G (p.Gln250Arg)	rs876660971	US
		*Exon 3*	c.766C>A (p.Leu256Met)	—	US
		*Exon 3*	c.778C>T (p.Arg260Trp)	rs121908150	P
		*Exon 3*	c.907G>A (p.Asp303Asn)	rs121908153	P
		*Exon 3*	c.910G>A (p.Glu304Lys)	rs180177484	P
		*Exon 3*	c.914T>C (p.Leu305Pro)	rs180177431	LP
		*Exon 3*	c.925G>C (p.Gly309Arg)	rs1057524777	LP
		*Exon 3*	c.926T>C (p.Phe309Ser)	rs121908154	P
		*Exon 3*	c.937A>G (p.Ile313Val)	rs180177501	US
		*Exon 3*	c.944C>T (p.Pro315Leu)	rs180177462	US/LB
		*Exon 3*	c.1027G>A (p.Glu343Lys)	rs369910640	US
		*Exon 3*	c.1043C>T (p.Thr348Met)	rs151344629	P
		*Exon 3*	c.1055C>T (p.Ala352Val)	rs121908149	P
		*Exon 3*	c.1058T>C (p.Leu353Pro)	rs28937896	P
		*Exon 3*	c.1070A>G (p.Lys357Arg)	rs876660972	US
		*Exon 3*	c.1071A>C (p.Lys357Asn)	rs1131691298	P
		*Exon 3*	c.1108A>C (p.Ile370Leu)	rs200735245	US
		*Exon 3*	c.1213A>C (p.Thr405Pro)	rs180177445	P
		*Exon 3*	c.1303A>G (p.Thr435Ala)	rs876661016	US
		*Exon 3*	c.1306A>G (p.Thr436Ala)	rs180177465	LP
		*Exon 3*	c.1316C>T (p.Ala439Val)	rs121908146	P
		*Exon 3*	c.1339C>T (p.Leu447Phe)	rs202121800	US
		*Exon 3*	c.1367G>A (p.Gly456Glu)	rs199696688	US
		*Exon 3*	c.1463G>A (p.Arg488Lys)	rs145268073	US/LB
		*Exon 3*	c.1631C>T (p.Thr544Met)	rs199856287	US
		*Exon 3*	c.1705G>C (p.Gly569Arg)	rs121908151	P
		*Exon 3*	c.1711G>A (p.Gly571Arg)	rs121908151	P
		*Exon 3*	c.1718T>C (p.Phe573Ser)	rs121908152	P
		*Exon 3*	c.1789A>G (p.Ser597Gly)	—	LP
		*Exon 3*	c.1805A>G (p.Gln602Arg)	rs1057518827	LP
		*Exon 3*	c.1845A>T (p.Lys615Asn)	rs876660973	US
		*Exon 3*	c.1880A>G (p.Glu627Gly)	rs121908148	P
		*Exon 3*	c.1942G>T (p.Asp648Tyr)	rs138061418	US
		*Exon 3*	c.2113C>A (p.Gln705Lys)	rs35829419	US
		*Exon 3*	c.2113C>A (p.Gln705Lys)	rs35829419	B
		*Exon 3*	c.2138A>T (p.His713Leu)	rs767805817	LB
		*Exon 4*	c.2182A>G (p.Ser728Gly)	rs147946775	US/LB
		*Exon 4*	c.2305G>A (p.Gly769Ser)	rs866534904	US
		*Exon 5*	c.2383A>G (p.Ser795Gly)	rs1064797023	US
		*Exon 5*	c.2398C>A (p.Leu800Met)	rs756392002	US
		*Exon 5*	c.2431G>A (p.Gly811Ser)	rs141389711	US
		*Exon 6*	c.2494C>A (p.Leu832Ile)	rs114158404	US
		*Exon 6*	c.2542G>C (p.Ala848Pro)	rs773376112	US
		*Exon 6*	c.2576A>G (p.Tyr859Cys)	rs180177452	P
		*Exon 6*	c.2617G>A (p.Ala873Thr)	rs201867582	US
		*Exon 6*	c.2638A>G (p.Lys880Glu)	rs1057515488	US
		*Exon 7*	c.2744C>T (p.Thr915Met)	rs765925466	US
		*Exon 7*	c.2759G>A (p.Arg920Gln)	—	P
		*Exon 7*	c.2767A>G (p.Thr923Ala)	rs200089542	US
		*Exon 7*	c.2790A>C (p.Lys930Asn)	rs876660975	US
		*Exon 7*	c.2825A>G (p.Lys942Arg)	rs201580005	US
		*Exon 8*	c.2861C>T (p.Thr954Met)	rs139814109	US/LB
		*Exon 8*	c.2895_2902del (p.(Ser966Profs^∗^10))	—	US
		*Exon 8*	c.2969G>C (p.Cys990Ser)	rs876660974	US
		*Exon 8*	c.2993G>C (p.Cys998Ser)	rs199517145	US
		*Exon 9*	c.3043A>G (p.Lys1015Glu)	rs771315000	US

List of abbreviations: P: pathogenic; LP: likely pathogenic; US: uncertain significance; LB: likely benign; B: benign.
